# The essential oil from the rhizomes of *Stahlianthus involucratus* attenuates the progression of vascular aging and atherosclerosis by regulating Nrf2-mediated mitochondrial quality

**DOI:** 10.3389/fphar.2025.1579333

**Published:** 2025-05-20

**Authors:** Jianmei Li, Zaishang Wang, Yu Wang, Jiaxin Lin, Ning Tang, Chu Zheng, Qin Xu

**Affiliations:** ^1^ College of Pharmacy, Guilin Medical University, Guilin, China; ^2^ Drug Dispensing Department, Zibo Central Hospital, Zibo, China; ^3^ Grain and Oil Pantry, Guangxi Guilin Institute for Food and Drug Control, Guilin, China

**Keywords:** atherosclerosis, Nrf2 signaling pathway, mitochondria, cardiovascular diseases, oxidative stress

## Abstract

**Introduction:**

Atherosclerosis (AS), characterized by chronic inflammation within the vasculature, is linked to endothelial dysfunction and oxidative stress. The senescence of vascular endothelial cells (VECs) serves as a pathological basis for AS. Stahlianthus involucratus (King ex Baker) Craib ex Loes is a folk medicinal plant commonly used in Guangxi has its rhizome as the active component. In traditional Chinese medicine, it is believed to promote blood circulation, remove blood stasis, stop bleeding, and disperse accumulated blood. Essential oil extracted from Stahlianthus involucratus rhizomes (EOSIR) is a key bioactive component. However, little research has been reported on the effects of EOSIR on cardiovascular disease.

**Methods:**

Validation techniques, including H&E staining and western blotting, were employed to assess the efficacy of EOSIR in both in vivo and in vitro models of AS.

**Results:**

In vivo experiments, EOSIR decreased plaque volume in atherosclerotic vessels of mice. Activation of the Nrf2 signaling pathway by EOSIR alleviated ox-LDL-induced injury in HUVECs, including a reduction in cellular senescence, apoptosis, ROS, and mitochondrial membrane potential, effects that were reversed by Nrf2 silencing. EOSIR also restored mitochondrial morphology in cells and enhanced Nrf2 expression as well as ATP levels in aortic tissue. Both in vivo and in vitro, EOSIR upregulated the Nrf2, NQO1, and HO-1 expression, downregulated Keap1 expression, and improved the mitochondrial-associated protein expression.

**Discussion:**

These findings suggest that EOSIR may prevent the onset of AS both in vivo and in vitro by modulating the mitochondrial quality control system through the Nrf2 signaling pathway.

## 1 Introduction

Cardiovascular disease (CVD) is a prevalent condition that represents a significant threat to human health, characterized by high morbidity, mortality, and prolonged treatment courses. Atherosclerosis (AS) occurs frequently in CVD and involves complex pathophysiological mechanisms, including oxidative stress, abnormal lipid metabolism, and impaired endothelial function (Yang et al., 2017).

Mechanisms associated with the senescence of vascular endothelial cells (VECs) play a crucial role in the development of AS. It has been proposed that oxidative stress induced by oxidized low-density lipoprotein (ox-LDL) in endothelial cells are closely associated with cellular senescence (Jiang et al., 2020). Senescent VECs show elevated production of reactive oxygen species (ROS), uncoupling of endothelial nitric oxide synthase (eNOS), and increased cellular permeability. These changes promote lipid infiltration and foam cell formation, disrupt hemostasis, and facilitate the persistence of microthrombi, collectively contributing to the progression of atherosclerotic (AS) plaques (Jia et al., 2019; Koutsaliaris et al., 2022).

A vast array of data points to the fact that the transcription of several antioxidant enzymes that either directly or indirectly control AS activities is activated by the nuclear factor E2-related factor 2 (Nrf2) signaling pathway. Activation of the Nrf2 signaling pathway, a critical regulator of antioxidant and anti-inflammatory responses, suppresses the progression of AS (Al-Mubarak et al., 2021; Gu et al., 2019). Consequently, it is of significant significance to further investigate the Nrf2 pathway and its targets in AS.

Mitochondria, the cellular “powerhouse”, are highly active organelles responsible for generating the energy required by the cell throughout its lifespan. The processes of mitochondrial biosynthesis, fission, fusion, and autophagy, which are collectively referred to as mitochondrial quality control, maintain mitochondria in a stable state (Hao et al., 2019). Studies have shown that dysregulated mitochondrial quality control can contribute to the aging of VECs. Excessive production of mitochondrial ROS (Mikhed et al., 2015), impaired mitochondrial biosynthesis (Dupre et al., 2019), altered mitochondrial dynamics (fusion and fission) (Jin et al., 2021), and defective mitochondrial autophagy (Ren and Zhang, 2018) are key intrinsic mechanisms that lead to senescence and dysfunction of VECs. Mitochondrial dysfunction, in turn, induces apoptosis and lipid alterations that contribute to the progression of atherosclerosis AS (Dromparis and Michelakis, 2013). Thus, dysregulated mitochondrial quality control is closely linked to the senescence of VECs and the progression of AS.

Traditional Chinese medicine (TCM) offers distinct benefits for treating and preventing CVDs. Most of the plants in the ginger family (Zingiberaceae) are perennial botanical drugs with aromatic properties and contain many well-known herbs and medicinal plants. EOFAZ, the oil derived from Fructus Alpinia zerumbet (FAZ), constitutes a principal active ingredient in FAZ of the Zingiberaceae family, which can protect endothelial cells from damage and has several pharmacological properties, like anti-inflammatory, antioxidant and anti-AS activity (Huang et al., 2017; Wang et al., 2024). *Stahlianthus involucratus* (King ex Baker) Craib ex Loes is a plant belonging to the Zingiberaceae family, primarily cultivated in the Guangxi and Yunnan regions. This species is a traditional Zhuang medicine in Guangxi folklore, renowned for its ability to disperse blood stasis, reduce swelling, alleviate pain, and stop bleeding (Wu et al., 2016). Additionally, its extracts have been demonstrated to decrease inflammatory reactions, alleviate oxidative stress, and inhibit ROS generation (Pingsusaen et al., 2015). The essential oil derived from *S. involucratus* rhizomes (EOSIR) is an oily mixture obtained from the dried rhizomes of *S. involucratus*. Currently, no study has reported on the protective efficacy of EOSIR in AS.

The research team previously demonstrated that EOSIR could alleviate ox-LDL-induced mitochondrial morphological damage and restore mitochondrial membrane potential in human umbilical vein endothelial cells (HUVECs). This effect is likely associated with the maintenance of mitochondrial homeostasis, suggesting that regulating mitochondrial mass might serve as a protective strategy for EOSIR in modulating VEC aging and the development of AS. On this basis, therefore, we used ox-LDL-stimulated human umbilical vein endothelial cells (HUVECs) as an *in vitro* model of AS and constructed the *in vivo* AS model by feeding ApoE^−/−^ knockout mice a high-fat diet. The research outcomes contribute experimental proof for the prevention and management of AS and associated CVDs through EOSIR and provide the basis for the further investigation and development of Guangxi Taoist medicinal plants and TCMs for treating CVDs.

## 2 Materials and methods

### 2.1 Reagents

Fetal Bovine Serum (FBS) was obtained from Competition (Guangzhou) Biotechnology Co., Ltd. (FBSAD-01011-500, Guangzhou, China). Dulbecco’s modified Eagle medium (DMEM) was obtained from Gibco (C11995500BT, New York, United States). Penicillin-Streptomycin Solution (PS) (100X), ATP Content Determination Kit, Mitochondrial Membrane Potential Assay Kit with JC-1 was sourced from Beijing Solarbio Science&Technology Co., Ltd. (P1400, BC0305, M8650, Beijing, China). Oxidized low-density lipoprotein (ox-LDL) were obtained from Dalian Meilun Biotechnology Co., Ltd. (MB12474, Dalian, China). ML385 (an Nrf2 inhibitor), and Bardoxolone Methyl (BARD) (an Nrf2 agonist) were obtained from MCE (HY-100523, SMB00376-10MG, New Jersey, United States). Lipofectamine 2000 was obtained from Invitrogen (11668027, California, United States). Senescence β-Galactosidase Staining Kit, NO Assay Kit, Reactive Oxygen Species Assay Kit, Mito-Tracker Green, and 4% Paraformaldehyde (PFA) Solution were obtained from Beyotime Biotechnology (C0602, S0021S, S0033S, C1048, P0099-500 mL, Shanghai, China). Enzyme-linked immunosorbent assay (ELISA) kits of ET-1, and PGI2 were obtained from Huamei Bioengineering Co., Ltd. (CSB-E07007h, CSB-E09591h, Wuhan, China).Primary antibodies (p53, p21, Keap1, Nrf2, NQO1, HO-1, DRP1, OPA1, MFN1, PGC1-α, TFAM, PINK1, LC3Ⅰ/Ⅱ, p62, Bax, Bcl-2, caspase-9, caspase-3) were obtained from Abcam (ab32389, ab109520, ab119403, ab62352, ab80588, ab305290, ab184247, ab157457, ab221661, ab313559, ab176558, ab216144, ab192890, ab109012, ab32503, ab182858, ab32539, ab32351, MA, United States). Primary antibodies (β-actin, GAPDH) and secondary antibodies (HRP-labeled Goat Anti-Mouse IgG, HRP-labeled Goat Anti-Rabbit IgG) were obtained from Cell Signaling Technology (3700, 97166, 7076, 7074, MA, United States). PCR primers were obtained from Wuhan Servicebio Technology Co., Ltd. (Wuhan, China).

*S. involucratus* (King) Craib (Lot: 110516) was obtained from Nanning Shennengtang Health Products Science and Technology Co., Ltd. (Nanning, China), and identified by Dr. Maoxiang Lai (Guangxi Institute of Chinese Medicine & Pharmaceutical Science).

### 2.2 Equipment

Flow cytometer (Canto plus, BD, United States), Transmission electron microscope (HT7700, Hitachi, Japan), Ultrathin microtome (electron microscopy) (EM UC7, Leica Microsystems, Germany), Chemiluminescence imaging system (ChemiDoc XRS+, BIO-RAD, United States), Ultra-speed freezing centrifuge (CP100NX, Hitachi, Japan), Real-time fluorescence quantitative PCR (ABI 7500 fast, Thermo Fisher Scientific, United States).

### 2.3 Preparation of EOSIR and gas chromatography mass spectrometry (GC-MS) analysis

The dried tubers of *S. involucratus* (King) Craib were chopped, soaked in distilled water, and left to fully hydrate for 3 h. The oil was extracted by steam distillation for 6 h. The volatile orange oil was then collected. The chemical constituents of EOSIR were analyzed by GC-MS (Agilent 6890NGC-5975MS, Agilent Technologies Inc., United States) with a DB-5MS capillary column. The mass spectra were compared with the NIST 2012 standard spectral library for identification.

### 2.4 Preparation of EOSIR emulsion

EOSIR (1.8, 0.9 and 0.45 mL) was dissolved in 5 mL of Tween-80, sonicated for 30 min and saline was taken up to a total volume of 50 mL to obtain EOSIR emulsion (75, 150 and 300 mg/kg).

### 2.5 Animal grouping and modeling methods

Forty-eight male ApoE^−/−^ mice (18–22 g, Guangdong Pharmachem Biotechnology Co., Ltd., SCXK (GD) 2020-0054) were housed under SPF conditions. The animal study protocols were reviewed and approved by the Experimental Animal Ethics Committee at Guilin Medical College (reference number GLMC-IACUC-2024013). Mice were divided into 6 groups (n = 8): control, high fat diet (HFD), EOSIR (75 mg/kg, 150 mg/kg, 300 mg/kg), positive control BARD (15 mg/kg). The experimental period was 12 weeks, during which mice were fed HFD (containing 22.5% fat and 0.15% cholesterol, Beijing Botaihongda Biotechnology Co., Ltd. (Beijing, China)) to induce AS, and controls were given a normal diet. Starting from the eighth week, the mice were gavaged daily with different doses of EOSIR and the positive control drug BARD. Saline was administered equally to both the control and HFD groups through gavage. The gavage volume was (0.1 mL/10 g) once a day for 4 weeks.

### 2.6 Specimen collection and paraffin section preparation

After the final drug treatment, the mice were fasted overnight but were not given water. They were then euthanized via intraperitoneal injection of sodium pentobarbital, and blood was collected by puncturing the heart. The hearts were perfused with pre-cooled saline through the left ventricle until the blood was completely washed out. Both the heart and aortic tissues were collected, frozen in liquid nitrogen, and kept at −80°C. 4% paraformaldehyde (PFA) fixation of aortic tissue, dehydrated, and cleared in xylene to render the tissue transparent. It was then sequentially immersed in paraffin wax (1, 2, and 3) for 1 h each. The paraffin-embedded tissue block was processed using a tissue embedding machine and a cryostat. Aortic paraffin sections were sliced into 4-μm-thick sections with a microtome, unfolded in water, and mounted onto adhesive slides.

### 2.7 Hematoxylin-eosin (H&E) staining

After deparaffinization, the paraffin sections were immersed in hematoxylin solution for 10 min. The addition of 1% hydrochloric acid followed, and the sections were subsequently reblued in 1% ammonia solution and washed with water. After eosin staining, the sections were immersed in xylene. After sealing the film, it was observed by a Fluorescent Inverted microscope (DMi8, Leica Microsystems, Germany) and photographed.

### 2.8 qRT-PCR

Total RNA was extracted from the samples using TRIzol reagent, and its concentration was quantified. Total RNA was reverse transcribed into cDNA using the FastKing Reverse Transcription Kit, and the mRNA levels of Keap1, Nrf2, HO-1, and NQO1 were quantified by the 2^−ΔΔCT^ method. The primer sequences are listed in Table 1.

**TABLE 1:** qRT-PCR Primers.

Gene	Forward primer	Reverse primer
Keap1	5′-GGG​CTT​TGA​GGG​ACT​AAC​C-3′	5′-ATC​CGC​CAC​TCA​TTC​CTC​TCT-3′
Nrf2	5′-TCC​AGT​CAG​AAA​CCA​GTG​GAT-3′	5′-GAA​TGT​CTG​CGC​CAA​AAG​CTG-3′
HO-1	5′-AAG​ACT​GCG​TTC​CGC​TCA​ACT-3′	5′-AAA​GCC​CTA​CAG​CAA​CTG​TCG-3′
NQO1	5′-TAT​CAC​CAC​TGG​GGG​TAG​CG-3′	5′-GGA​GTG​TGG​CCA​ATG​CTG​TAA-3′
GAPDH	5′-CCT​CGT​CCC​GTA​GAC​AAA​ATG-3′	5′-TGA​GGT​CAA​TGA​AGG​GGT​CGT-3′

### 2.9 Cell culture

Cell lines as well as ox-LDL (200 μg/mL) and EOSIR (0.05 μg/mL) were selected based on previous studies. HUVECs were purchased from Shanghai Aolu Biotechnology Co. Ltd. (Shanghai, China) and cultured in DMEM containing 10% FBS, 1% PS at 37°C with 5% CO2. The fractions were: control, ox-LDL, ox-LDL + EOSIR, ox-LDL + EOSIR + ML385, ox-LDL + ML385, ox-LDL + BARD, ox-LDL + EOSIR + si-Nrf2, ox-LDL + EOSIR + si-NC. In effect, cells were co-treated with 200 μg/mL ox-LDL or 200 μg/mL ox-LDL with 0.05 μg/mL EOSIR in the ox-LDL group for 24 h. To delve deeper into whether ox-LDL-induced cellular damage is linked to the Nrf2 signaling pathway, we co-treated ox-LDL-treated cells with 20 μM ML385 or 50 nM BARD for 24 h. Additionally, HUVECs were transiently transfected with si-Nrf2 to knock down Nrf2 expression.

### 2.10 Transient transfection with siRNA

HUVECs were transfected with 50 nM Nrf2 siRNA using Lipofectamine 2000. The transfection medium was serum- and antibiotic-free. After 8 h of transfection, the medium was replaced with semi-complete medium containing 10% FBS, and incubation was extended by 12 h before drug exposure. The si-Nrf2 sequences are listed in Table 2.

**TABLE 2:** si-Nrf2 sequences.

Gene	Sense	Antisense
si-Nrf2a	5′-GCC​UGU​AAG​UCC​UGG​UCA​UTT-3′	5′-AUG​ACC​AGG​ACU​UAC​AGG​CTT-3′
si-Nrf2b	5′-GCC​CAU​UGA​UGU​UUC​UGA​UTT-3′	5′-AUC​AGA​AAC​AUC​AAU​GGG​CTT-3′
si-Nrf2c	5′-GGG​AGG​AGC​UAU​UAU​CCA​UTT-3′	5′-AUG​GAU​AAU​AGC​UCC​UCC​CTT-3′

### 2.11 β-galactosidase (SA-β-gal) staining

HUVECs suspensions were cultured in 24-well plates at a density of 3 × 10^4^ cells/mL for 24 h. After then, ox-LDL, EOSIR, ML385, or BARD were added, and the cells were co-treated for 24 h. The culture medium was then removed. Each well was fixed with 250 μL of fixative for 15 min. Following fixation, 250 μL of staining solution was applied, and the cells were stained for 12 h at 37°C, protected from light. Finally, the cells were observed under an inverted microscope.

### 2.12 Determination of NO level

HUVECs were treated following method 2.6, and the supernatant was collected. The samples and standards were analyzed according to the manufacturer’s instructions. Standard curves were generated, and the NO concentrations for each group were calculated.

### 2.13 ELISA

Following treatment as described in Method 2.6, the supernatant of HUVECs was collected. ET-1 and PGI2 levels were measured according to the kit instructions, and a standard curve was constructed using standard dilutions to calculate the concentration of each sample.

### 2.14 Reactive oxygen assay

After treatment according to method 2.6, 10 μM/L of DCFH-DA was incubated with HUVECs in an incubator for 20 min, followed by washing. Observed under inverted fluorescence microscope and photographed.

### 2.15 Mitochondrial membrane potential detection

After performing the treatment as described in method 2.6, 250 μL of cell culture medium and JC-1 staining solution were added sequentially. The cells were then incubated for 20 min in an incubator, washed twice with a specialized washing buffer, and subsequently observed under an inverted fluorescence microscope for imaging.

### 2.16 Mitochondrial membrane potential detection

The cells were seeded into confocal dishes, grouped, and processed. Then, HUVECs were incubated with the prepared Mito-Tracker Green staining solution in an incubator for 30 min. Observations were performed and images were captured using a laser-scanning confocal fluorescence microscope (CLSM) (LSM710, Carl Zeiss AG, Germany).

### 2.17 Flow cytometry

HUVECs were seeded in suspension at a density of 4 × 10^5^ cells/mL in 60 mm culture dishes and incubated for 24 h. After the treatment, cells were harvested, washed, and resuspended in 500 µL of binding buffer. The cells were dark-treated with 5 µL Annexin V-FITC and/or 10 µL PI for 15 min at room temperature and assayed on the machine.

### 2.18 Mitochondrial membrane potential detection

The cell samples were fixed with 4% PFA for 20 min and then permeabilized using a permeabilization solution for 20 min. Tissue samples underwent antigen retrieval in the microwave, followed by natural cooling, washing, and drying. The samples were blocked with a solution containing 3% bovine serum albumin (BSA) for 30 min. The primary antibody (Nrf2, 1:400) was added dropwise to the samples and incubated overnight at 4°C. Following this, the samples were incubated with the secondary antibody at room temperature for 1 h, protected from light. The samples were blocked with drops of anti-fluorescence quenching agent (containing DAPI) and observed and photographed under an inverted fluorescence microscope.

### 2.19 Transmission electron microscopy (TEM)

The cells were treated according to their respective drug administration groups and then fixed with 2.5% glutaraldehyde for 18–20 h. Subsequently, 1 mL of PB containing 5% BSA was added to each well. The adherent cells were harvested by scraping with a cell scraper, collected into a centrifuge tube, and centrifuged. The cells were then fixed with 1% osmium tetroxide, stained with uranyl acetate, dehydrated using a graded ethanol series, and infiltrated with acetone at varying concentrations. The resins were impregnated overnight, embedded in a mold, polymerized, and subsequently made into ultrathin sections. TEM analysis was performed to assess mitochondrial morphology, and images were captured and analyzed.

### 2.20 Determination of ATP content

The sample was added in proportion to the ATP extract, extracted in an ice bath, and then centrifuged at low temperature and high speed. The supernatant was collected. Chloroform was added, and the mixture was shaken well before being centrifuged again. The resulting supernatant is the desired sample. The samples and standards were tested according to the kit instructions, and the ATP content of each group was calculated using the provided formula.

### 2.21 Western blot (WB)

Total protein of the samples was collected with RIPA lysate containing 1% PMSF, and protein concentration was subsequently determined. Proteins were separated by SDS-PAGE, transferred to PVDF membranes, blocked with 5% skim milk, and incubated overnight at 4°C with different primary antibody ratios (1:1000 to 1:10 000). Secondary antibodies of the same species as the primary antibody were incubated for 1 h at room temperature. ECL reagent was dropped, and the signal was detected using a gel imaging system. Protein intensity was measured using ImageJ software.

### 2.22 Statistical analysis

Statistical analysis was performed using GraphPad Prism 8.0.2. Data are presented as the mean ± standard deviation (SD) from three independent experiments. Statistical differences were determined using a one-way ANOVA or a t-test. A P-value of less than 0.05 was considered statistically significant.

## 3 Results

### 3.1 Main components of EOSIR

**FIGURE 1:**
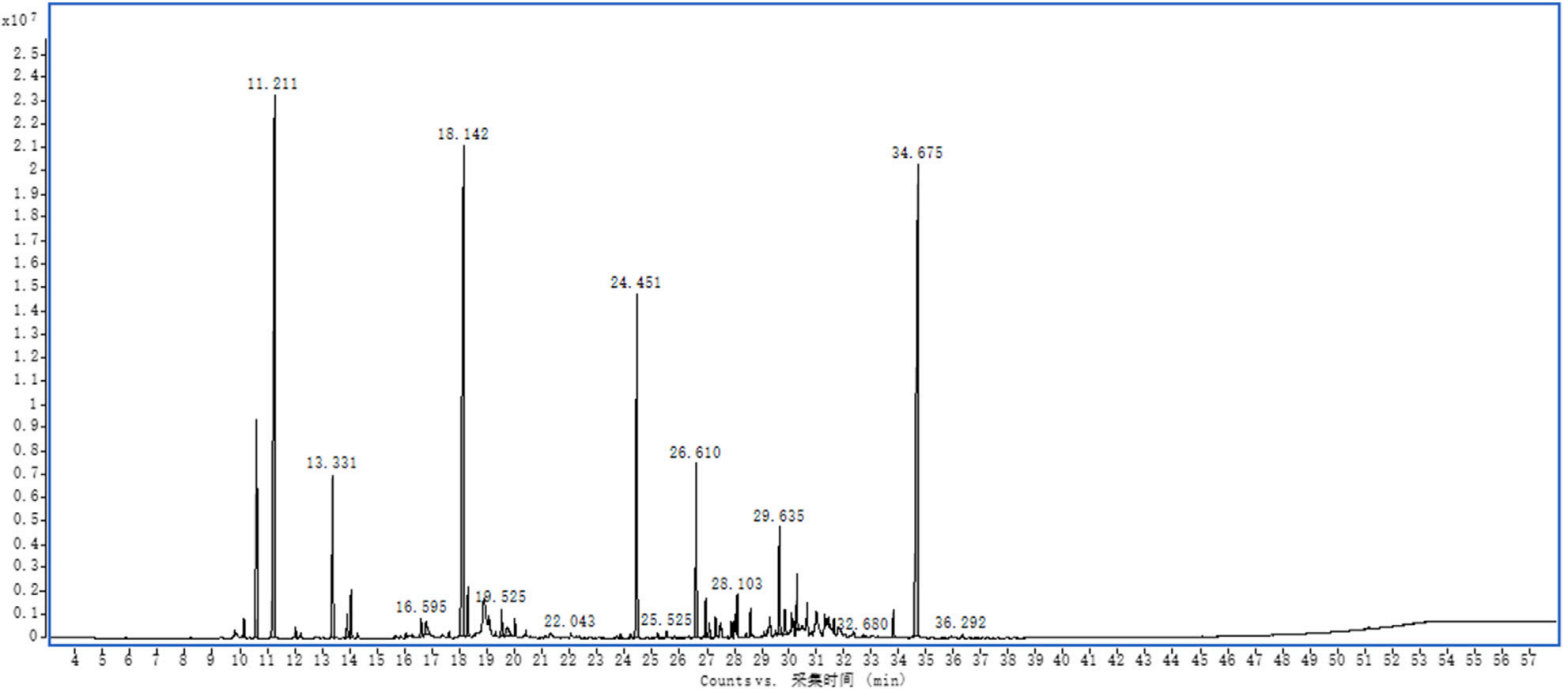
Fingerprint analysis of EOSIR by GC-MS.

EOSIR was determined using GC-MS, and the relative abundance was determined through the area normalization method. A total of 64 components were detected, with the top three components being 3,6,7,8-tetrahydro-3,3,6,6-tetramethyl-as-Indacen-1(2H)-one, (+)-2-Bornanone, camphene, which accounted for 20.52%, 19.49%, and 18.51%, respectively. (Supplementary Table S1; Figure 1).

### 3.2 EOSIR effects of EOSIR on aortic plaque formation and vascular senescence in AS mice

**FIGURE 2:**
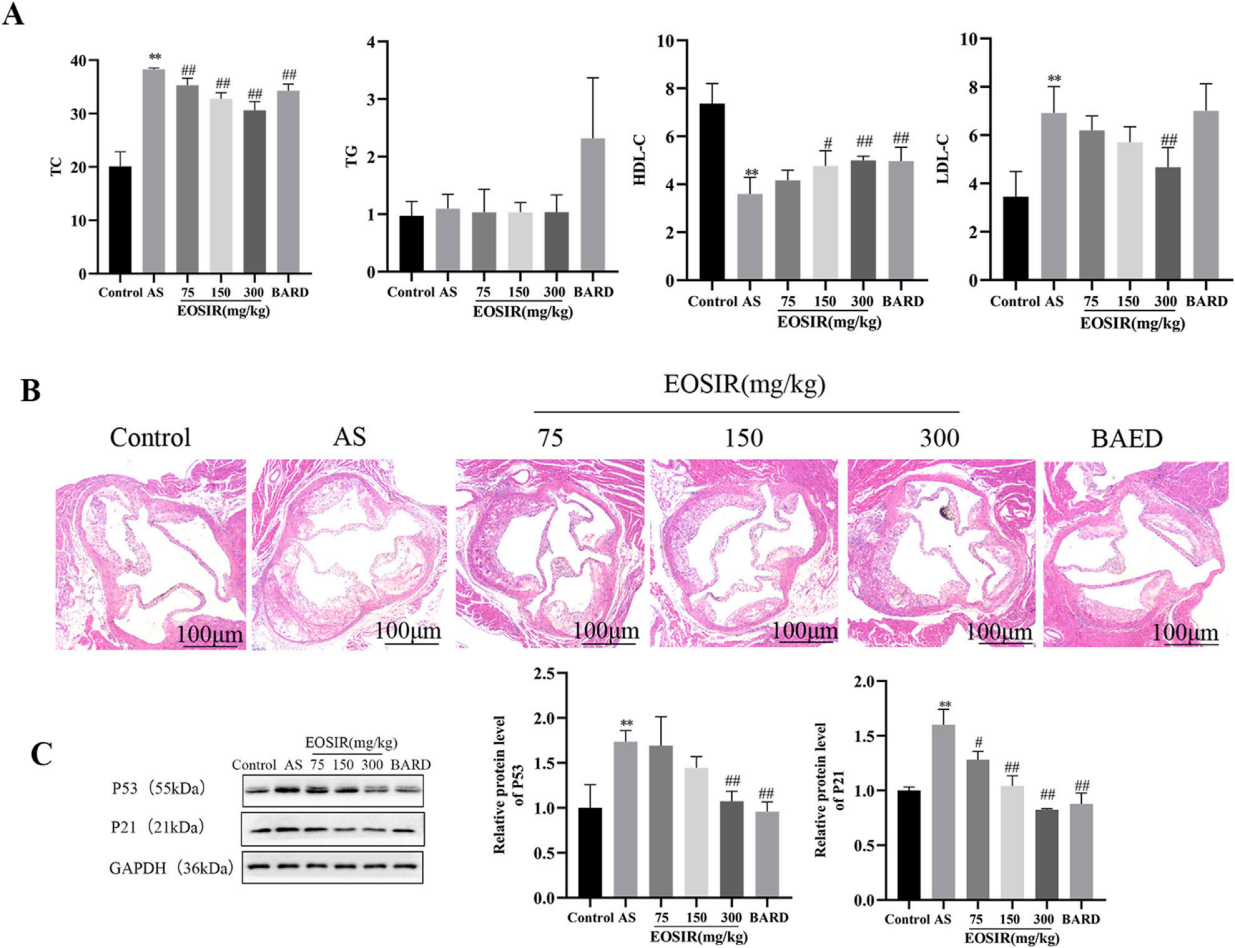
Effects of EOSIR on aortic plaque formation and vascular senescence in AS mice. **(A)** Lipid levels in mice (n = 8). **(B)** H&E staining of mouse aorta (x40) (n = 3). **(C)** WB assays for p53 and p21 expression(n = 3). ( $\bar{x}$ ±SD) (^**^P < 0.01 vs. control group; ^#^P < 0.05 or ^##^P < 0.01 vs. HFD group).

Abnormally elevated plasma cholesterol and triglyceride levels are significant pathogenic risk factors for AS (Beverly and Budoff, 2020). Very low-density lipoprotein (VLDL) and low-density lipoprotein (LDL) transport cholesterol and other components to the arterial intima, where they contribute to lipid accumulation and pathological deposition, ultimately leading to the formation of early lesions in AS (Ference et al., 2018). In this study, we analyzed four distinct lipid profiles in mice, and it was found that total cholesterol (TC), high-density lipoprotein (HDL-C), and low-density lipoprotein (LDL-C) were significantly different in mice maintained on only HFD compared with those on a normal diet. After gavaging the mice with different concentrations of EOSIR, the levels of TC, HDL-C, and LDL-C with increasing concentrations of EOSIR showed a significant trend. Specifically, HDL-C gradually increased, while TC and LDL-C gradually decreased. BARD also showed a similar trend. However, no statistically significant differences were observed in triglyceride (TG) with either HFD and/or EOSIR treatment (Figure 2A). HE staining of the aortas from each group of mice showed that the aortic root in the model mice contained numerous lipid plaques, and the arterial intima was significantly thickened with a large plaque volume. After EOSIR treatment, the aortic plaque area decreased, with the most notable therapeutic effect seen in mice fed 300 mg/kg EOSIR (Figure 2B). The formation and progression of lesions in AS are closely linked to cellular senescence. Endothelial cell senescence leads to alterations in vascular structure and function, disrupts angiogenesis and vascular integrity, and contributes to the worsening of atherosclerosis (Jia et al., 2019). WB analysis was used to detect senescence-associated proteins within the aortic tissue of mice across all groups. The results indicated that EOSIR and BARD inhibited the increase in p53 and p21 expression in mice induced by HFD (Figure 2C). These findings suggest that EOSIR slows the formation of aortic plaques as well as vascular senescence in HFD-induced AS mice.

### 3.3 Impact of EOSIR on the mitochondrial quality control system in AS mice

**FIGURE 3:**
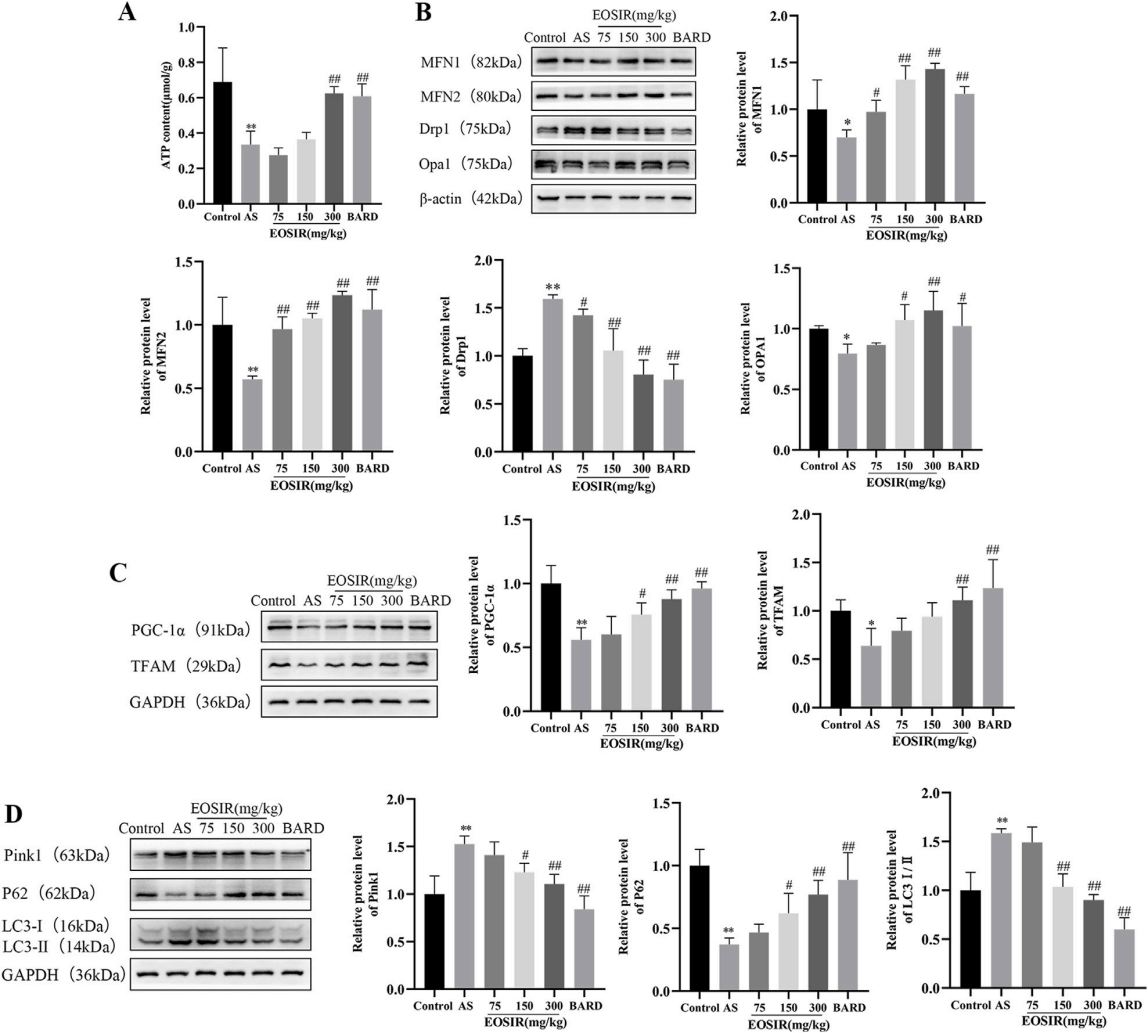
Impact of EOSIR on the mitochondrial mass control system in AS mice. **(A)** Microscopic analysis of ATP content in the mouse aorta(n = 8). **(B)** WB assays for MFN1, MFN2, Opa1 and Drp1 expression(n = 3). **(C)** WB assays for TFAM and PGC-1α expression(n = 3). **(D)** WB assays for p62, LC3Ⅰ/Ⅱ and Pink1 expression(n = 3). ( $\bar{x}$ ±SD) (^*^P < 0.05 or ^**^P < 0.01 vs. control group; ^#^P < 0.05 or ^##^P < 0.01 vs. HFD group).

ATP levels in mouse aortic tissues were detected using a micro-method to evaluate mitochondrial function. It was found that ATP levels were significantly reduced in AS mice than in control mice. In contrast, after 300 mg/kg EOSIR and BARD interventions, ATP levels were significantly elevated (Figure 3A).

Imbalance between mitochondrial fusion and fission results in the accumulation of damaged and dysfunctional mitochondria, leading to mitochondrial dysfunction. Mitochondrial fusion is primarily regulated by MFN1, MFN2, and Opa1(Poznyak et al., 2020). Mitochondrial fission aids in the removal of damaged mitochondria, with Drp1 serving as a key regulator of this process. Elevated Drp1 expression promotes mitochondrial fission and contributes to endothelial damage (Huang et al., 2018). We used WB to detect mitochondria-associated proteins in mouse aortic tissues and found that EOSIR upregulates the level of mitochondrial fusion proteins MFN1, MFN2, and Opa1, while inhibiting the abnormal increase of the mitochondrial fission protein Drp1 in the AS mice (Figure 3B).

Mitochondrial biogenesis is a complex, multistep process that governs mitochondrial DNA replication and gene expression. PGC-1α acts as a transcriptional activator of mitochondrial biogenesis, regulating the transcription of downstream genes, such as the mitochondrial transcription factor TFAM, both in the nucleus and in the mitochondria (Kadlec et al., 2016; Yao et al., 2016). In the present study, the reduced levels of the mitochondrial transcription factor TFAM and the mitochondrial biosynthesis factor PGC-1α in AS mice were reversed following intervention with EOSIR (Figure 3C).

Autophagy is a process in which long-lived proteins and functionally impaired or dysfunctional mitochondria are degraded by lysosomes. Basal autophagy plays a crucial role in maintaining cellular stability and overall organismal homeostasis (Martinet and De Meyer, 2008). It was observed that EOSIR treatment was able to improve the downregulation of p62 and reduce the elevated levels of Pink1 and LC3Ⅰ/Ⅱ, two proteins associated with mitochondrial autophagy (Figure 3D). Taken together, this experiment demonstrates that EOSIR is essential for inhibiting mitochondrial damage and regulating mitochondrial quality control.

### 3.4 Impact of EOSIR on the Nrf2 signaling pathway in AS mice

**FIGURE 4:**
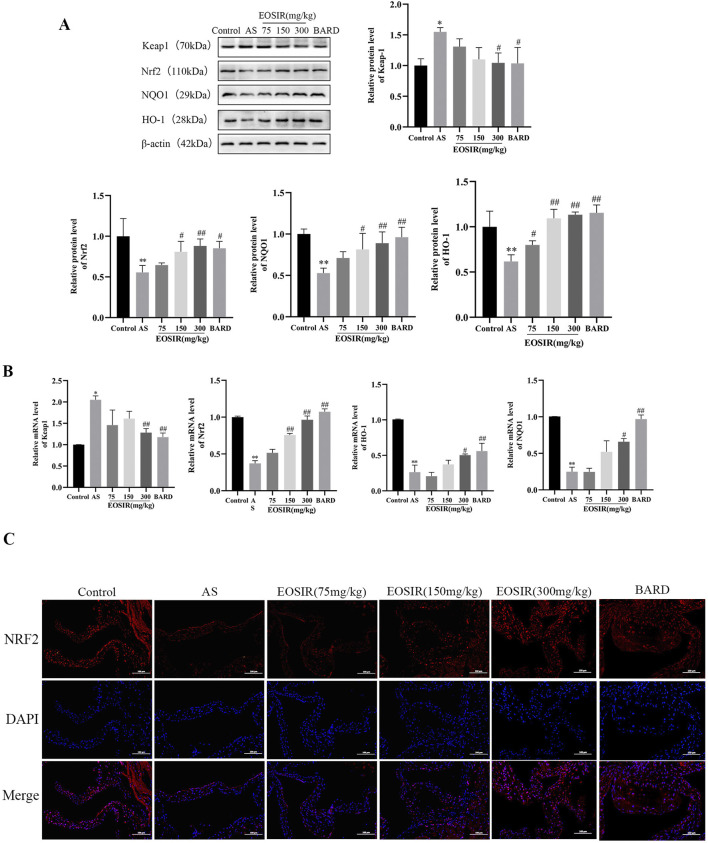
Impact of EOSIR on the Nrf2 signaling pathway in AS mice. **(A)** WB assays for Keap1, Nrf2, NQO1, and HO-1 expression. **(B)** qRT-PCR assays of Keap1, Nrf2, NQO1, and HO-1 mRNA expression. **(C)** Immunofluorescence staining of Nrf2 in mouse aortic tissue ( $\bar{x}$ ±SD, n = 3) (^*^P < 0.05 or ^**^P < 0.01 vs. control group; ^#^P < 0.05 or ^##^P < 0.01 vs. HFD group).

Under normal physiological conditions, Nrf2 is kept in the cytoplasm through its endogenous inhibitor Keap1. Upon oxidative stress, a conformational change in Keap1 triggers the release and subsequent translocation of Nrf2 to the nucleus, where it binds to the Antioxidant Response Element (ARE). This binding activates the expression of key antioxidant enzymes, including HO-1 and NQO-1, thereby providing a defense against oxidative damage (Yan et al., 2019; Kattoor et al., 2017). WB (Figure 4A) and qPCR (Figure 4B) were employed to examine the level of Keap1, Nrf2, and HO-1 and NQO1 in the aortic tissues of AS mice, determining whether EOSIR exerts beneficial effects on AS mice through the Nrf2 pathway in the *in vivo* experiments. The data revealed that the protein and mRNA expression of Nrf2 and its downstream antioxidant genes HO-1 and NQO1 were upregulated, whereas that of Keap1 was downregulated in the mouse aorta after HFD + EOSIR intervention, compared to HFD-only treated mice. Moreover, the positive drug BARD exhibited a similar trend of change as EOSIR. Furthermore, to further investigate the expression of Nrf2 in the aortic tissues of mice, immunofluorescence experiments revealed that Nrf2 expression was remarkably decreased in HFD-induced AS mice in contrast to untreated mice. However, a notable upregulation of Nrf2 expression was observed following EOSIR treatment (Figure 4C). In conclusion, EOSIR may be able to regulate the Keap1/Nf2/ARE pathway to exert a beneficial effect on HFD-induced AS mice.

### 3.5 EOSIR alleviates ox-LDL-induced senescence and functional impairment in HUVECs

**FIGURE 5:**
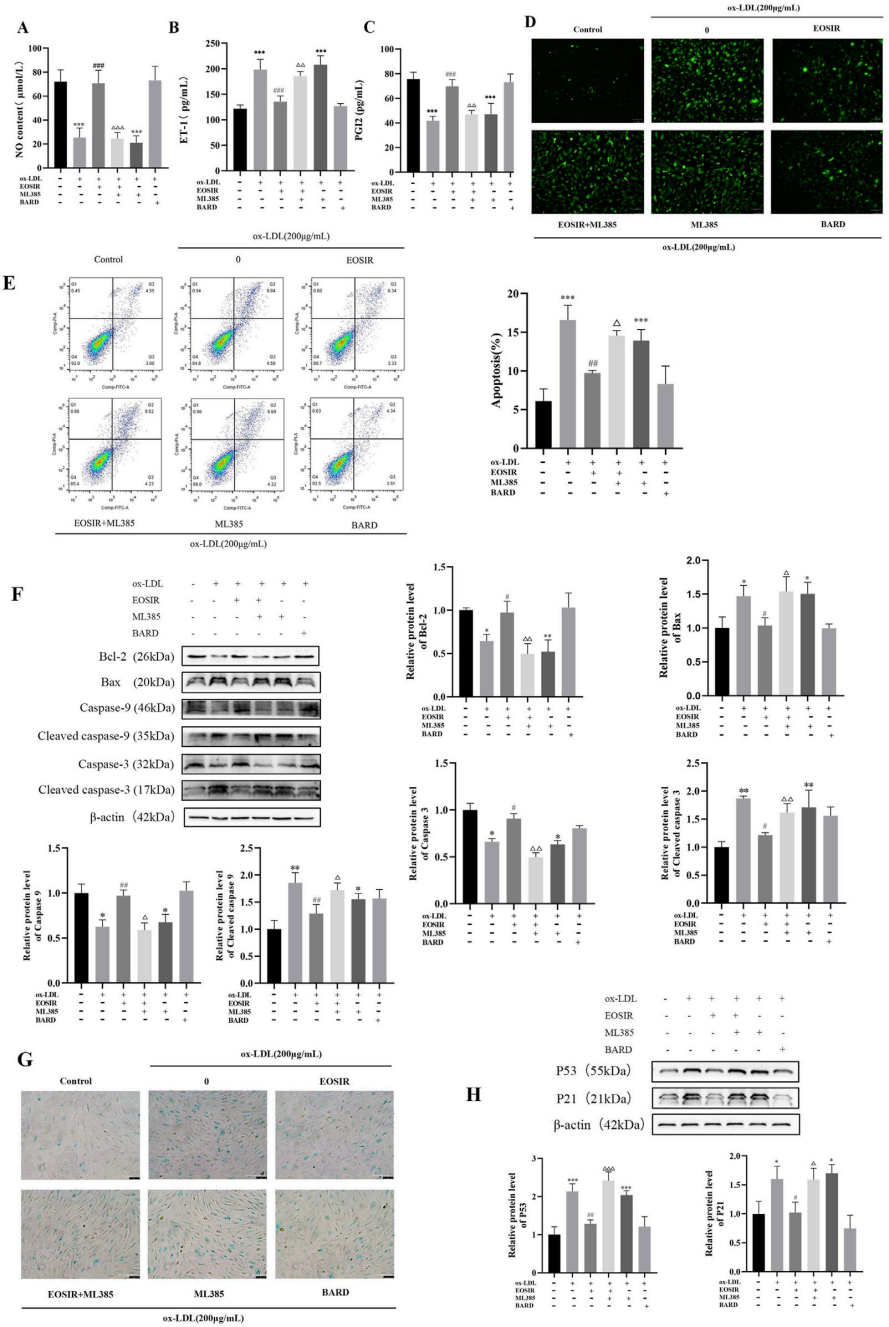
EOSIR alleviates ox-LDL-induced senescence and functional impairment in HUVECs. **(A)** Measurement of NO content. **(B)** Measurement of PGI2 levels. **(C)** Measurement of ET-1 levels. **(D)** Cellular ROS levels detected using the fluorescent probe DCFH-DA. **(E)** Flow cytometry for apoptosis detection. **(F)** WB assays for Bax, Bcl-2, caspase-9, caspase-3, cleaved caspase-9 and cleaved caspase-3 expression. **(G)** SA-β-gal staining. **(H)** WB assays for p53 and p21 expression.( $\bar{x}$ ±SD, n = 3) (^*^P < 0.05 or ^**^P < 0.01 or ^***^P < 0.001 vs. control group; ^#^P < 0.05 or ^##^P < 0.01 vs. ox-LDL group;^△^P < 0.05 or ^△△^P < 0.01 or ^△△△^P < 0.001 vs. EOSIR group).

The homeostasis of vascular endothelial function relies on the dynamic equilibrium of vasoactive substances. In this balance, NO and PGI2 serve a protective role by promoting vasodilation and inhibiting platelet aggregation, while abnormally ET-1 exacerbates vasoconstriction and inflammation. Additionally, ox-LDL contributes to endothelial dysfunction by triggering excessive production of ROS, inhibiting eNOS activity, and disrupting the PGI2/ET-1 balance (Chirkov et al., 2022; Yang et al., 2019). First, the detection of vasoactive substances in the supernatant revealed EOSIR significantly ameliorated the ox-LDL-treated reduction of NO and PGI2 and elevation of ET-1 when co-cultured with HUVECs, an effect that was counteracted by ML385 (Figures 5A–C). Subsequently, changes in ROS levels in HUVECs, both before and after EOSIR treatment, were assessed using the fluorescent probe DCFH-DA (Figure 5D). Results showed that intracellular levels of ROS were significantly higher after ox-LDL treatment. ROS levels decreased following EOSIR treatment, whereas inhibition of Nrf2 diminished the therapeutic effect of EOSIR. The evidence pointed to the fact that EOSIR can improve the concentration of vasoactive substances and ROS in HUVECs.

It is widely recognized that Bax and Bcl-2 are crucial regulators of the intrinsic apoptotic pathway, with Bcl-2 primarily inhibiting apoptosis without influencing the cell cycle or differentiation, whereas Bax promotes apoptosis (Ahmed et al., 2019). Apoptosis was assessed by flow cytometry (Figure 5E), revealing a significantly higher apoptosis rate in ox-LDL-induced HUVECs compared to that of the control normal HUVECs. Notably, EOSIR administration significantly decreased the apoptosis rate in HUVECs. To determine whether EOSIR affects apoptosis marker proteins in HUVECs, WB assayed the expression of Bax and Bcl-2 in HUVECs. According to, the expression of Bax and Bcl-2 was reduced and elevated, respectively, in ox-LDL + EOSIR co-treated HUVECs compared to HUVECs treated with ox-LDL alone (Figure 5F). And co-treatment with ox-LDL, EOSIR, and ML385 reversed the anti-apoptotic effect of EOSIR. It indicated that EOSIR can inhibit the overexpression of the apoptosis inhibitor Bax and reduce the suppressive effect of the pro-apoptotic factor Bcl-2 in HUVECs induced by ox-LDL, thereby mitigating the apoptosis of endothelial cells. The mitochondrial-mediated apoptotic pathway is mainly managed through cytochrome c delivery and caspase-9 activation, where Cytochrome c (Cyt c) activates the initiator caspase-9 to produce apoptotic bodies, which subsequently leads to the activation of caspase-3, ultimately leading to apoptotic cell death (Frión-Herrera et al., 2015). We also detected the expression of caspase-9, caspase-3, cleaved caspase-9 and caspase-3 in HUVECs by WB, and Figure 5F shows that after EOSIR intervention, the expression levels of caspase-9 and caspase-3 proteins were increased, and the expression levels of cleaved caspase-9 and cleaved caspase-3 were decreased. It suggests that EOSIR may improve apoptosis through the mitochondrial pathway.

Cellular senescence is a key feature of endothelial dysfunction in the atherosclerotic process, contributing to cell cycle arrest and altered secretory phenotypes. The hallmark characteristics of cellular senescence include elevated SA-β-gal activity and the activation of the p53-p21 signaling pathway (Fielder et al., 2022; Gao et al., 2016). The SA-β-gal staining kit was utilized to evaluate cellular senescence (Figure 5G). Data showed that following ox-LDL-induced cell injury, the proportion of blue-stained cells (senescence-positive), which indicates cellular senescence, increased. Moreover, the proportion of unstained cells (senescence-negative) increased after EOSIR treatment. WB analysis (Figure 5H) was conducted to evaluate p53 and p21 expression in HUVECs to assess whether EOSIR has an anti-aging effect in HUVECs injury. Compared to ox-LDL-induced HUVECs, p53 and p21 were significantly reduced in HUVECs treated with EOSIR. Furthermore, this effect was reversed when co-treated with ML385. These results clearly indicate that activation of Nrf2 by EOSIR alleviates ox-LDL-induced cellular senescence and functional impairment.

### 3.6 Impact of EOSIR on the mitochondrial quality control system of ox-LDL-induced HUVECs

**FIGURE 6:**
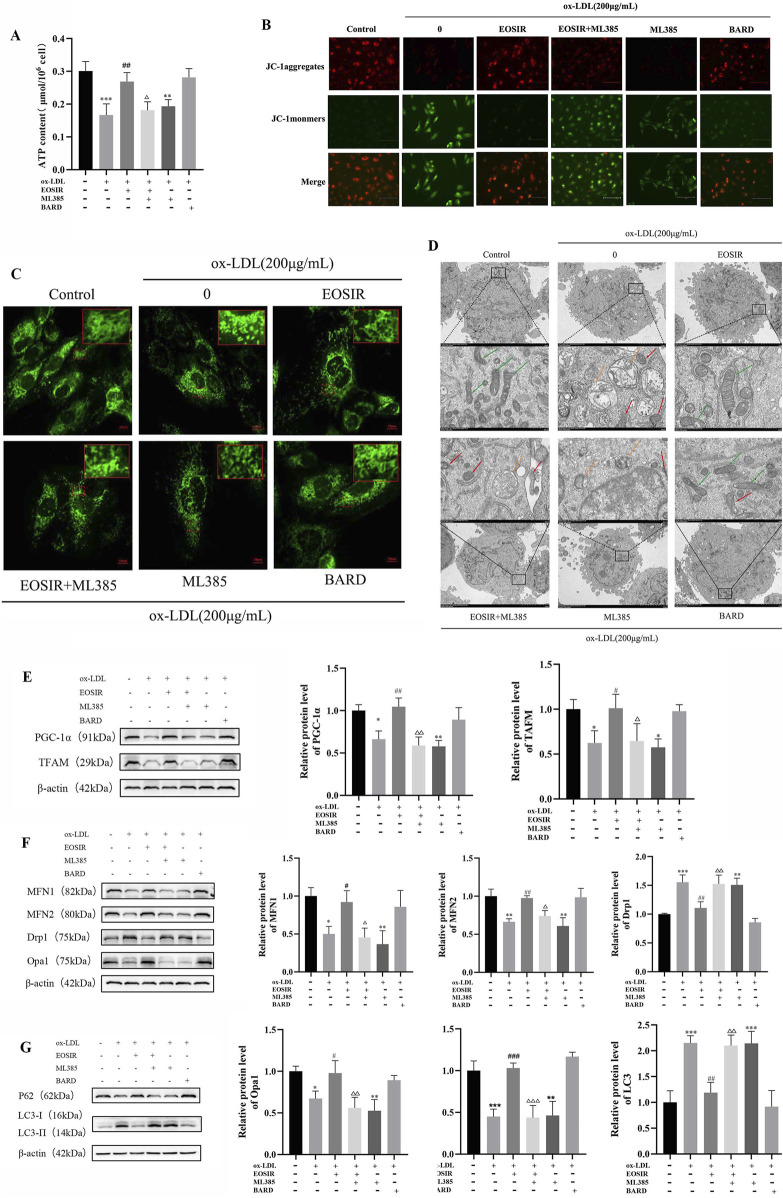
Impact of EOSIR on the mitochondrial quality control system of ox-LDL-induced HUVECs. **(A)** A micro-assay for detecting intracellular ATP content. **(B)** JC-1 staining for MMP. **(C)** Mito-Tracker Green staining to assess mitochondrial mass. **(D)** TEM imaging to observe mitochondrial morphology and autophagy. Green arrows indicate normal mitochondria, yellow arrows indicate autophagic vesicles, and red arrows indicate damaged mitochondria. **(E)** WB assays for MFN1, MFN2, Drp1, and Opa1 expression. **(F)** WB assays for TFAM and PGC-1α expression. **(G)** WB assays for LC3Ⅰ/Ⅱ and P62 expression. ( $\bar{x}$ ±SD, n = 3) (^*^P < 0.05 or ^**^P < 0.01 or ^***^P < 0.01 vs. control group; ^#^P < 0.05 or ^##^P < 0.01 vs. ox-LDL group;^△^P < 0.05 or ^△△^P < 0.01 or ^△△△^P < 0.001 vs. EOSIR group).

As the primary site of cellular energy metabolism, the ATP production capacity of mitochondria serves as a crucial indicator of mitochondrial function. It was significantly decreased following ox-LDL treatment of HUVECs compared to untreated HUVECs. Incubation of EOSIR with HUVECs restored intracellular ATP levels, which were significantly diminished upon Nrf2 inhibition (Figure 6A). Mitochondrial membrane potential (MMP) refers to the difference in electrochemical potential within the inner mitochondrial membrane, which is vital in maintaining mitochondrial function and energy production. The JC-1 dye is used to quantify the depolarization or hyperpolarization of the mitochondrial membrane. In its monomeric form, JC-1 emits green fluorescence; however, in active mitochondria, it aggregates into red-fluorescent structures known as J-aggregates. A higher red-to-green fluorescence ratio indicates a higher MMP (Sabirova et al., 2025). The results showed that MMP remained at normal levels in the control group, as evidenced by the red fluorescence produced by the aggregation of JC-1. However, after exposure to ox-LDL, there was a noticeable enhancement of green fluorescence and a weakening of red fluorescence, suggesting that JC-1 predominantly existed in its monomeric form. This indicates a significant reduction in MMP. However, treatment with EOSIR effectively reversed the increase in green fluorescence intensity, a marker of mitochondrial damage caused by ox-LDL (Figure 6B).

Studies have shown that cells with high metabolic activity and energy demands typically possess a highly interconnected mitochondrial network, and this flexible network enhances respiratory function by promoting the exchange of mitochondrial components (McCarron et al., 2013). Mitochondrial meshwork was visualized using LSCM following Mito-Tracker Green staining. In the control group, normal mitochondria formed an interconnected network within the cell, exhibiting a meshwork-like structure. In contrast, the mitochondrial network after ox-LDL stimulation of HUVECs was disrupted, with mitochondria becoming fragmented and dispersed. However, after incubation of EOSIR with HUVECs resulted in a reduction of mitochondrial fragmentation, an increase in mitochondrial length, and a partial restoration of the meshwork structure (Figure 6C). The TEM results revealed that in the control group, the cell nucleus, mitochondria, and other organelles appeared normal. In contrast, the model group exhibited autophagosomes with a double-layer membrane structure surrounding the cell nuclei, as well as mitochondria with noticeable loss of cristae and other morphological abnormalities. However, EOSIR intervention significantly decreased the number of ox-LDL-induced autophagosomes in HUVECs, and the organelle morphology was largely restored to normal (Figure 6D).

After the administration of ML385, the expression levels of mitochondria-related proteins were evaluated. EOSIR treatment led to a notable rise in the mitochondrial fusion proteins MFN1, MFN2, and OPA1, but a marked downturn in the mitochondrial fission protein Drp1, when compared with the ox-LDL group (Figure 6E). Analyzing mitochondrial biogenesis proteins further demonstrated that EOSIR effectively reversed the ox-LDL-induced reduction in TFAM and PGC-1α expression (Figure 6F). Analysis of autophagy-related proteins revealed LC3 Ⅰ/Ⅱ and p62 expression were reduced and elevated, respectively, in the ox-LDL + EOSIR group (Figure 6G). Furthermore, the beneficial effects of EOSIR observed earlier were diminished after ML385 treatment, suggesting that protective effects of EOSIR on mitochondrial damage and the mitochondrial quality control system was also attenuated following Nrf2 inhibition.

### 3.7 Effect of EOSIR on ox-LDL-induced Nrf2 signaling activation in HUVECs

**FIGURE 7:**
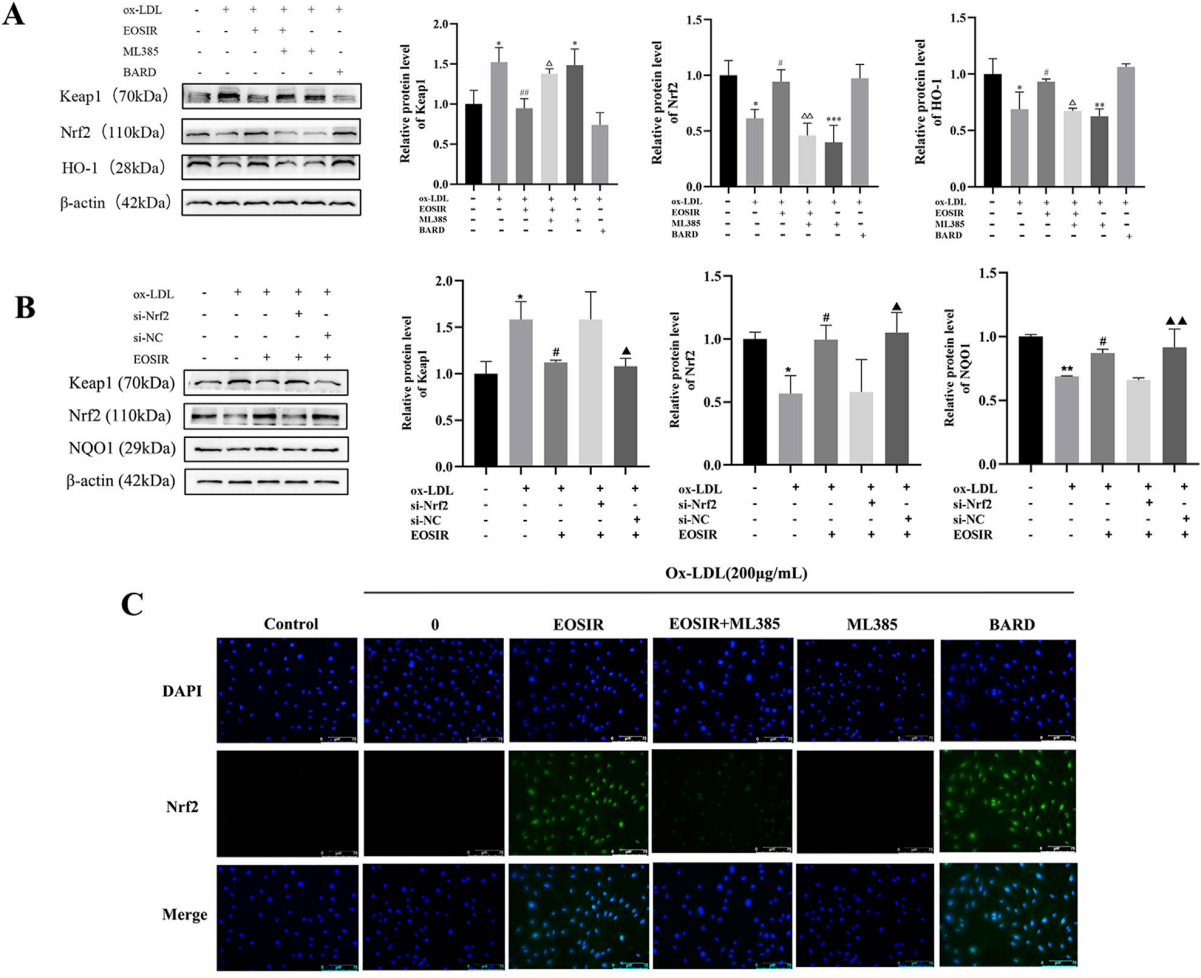
Effect of EOSIR on ox-LDL-induced Nrf2 signaling activation in HUVECs. **(A)** WB assays for Keap1, Nrf2, and NQO1 expression following Nrf2 inhibition. **(B)** WB assays for Keap1, Nrf2, and NQO1 expression after Nrf2 silencing. **(C)** Immunofluorescence staining of ox-LD-induced Nrf2 nuclear translocation in HUVECs. ( $\bar{x}$ ±SD, n = 3) (^*^P < 0.05 or ^**^P < 0.01 or ^***^P < 0.001 vs. control group; ^#^P < 0.05 or ^##^P < 0.01 vs. ox-LDL group;^△^P < 0.05 or ^△△^P < 0.01 vs. EOSIR group;^▲^P < 0.05 or ^▲▲^P < 0.01 vs. si-Nrf2 group).

To assess the role of the Nrf2 pathway in EOSIR-induced cellular injury to ox-LDL, we incubated HUVEC with Nrf2 inhibitors. Keap1 levels were significantly lower after EOSIR treatment compared to ox-LDL-stimulated HUVEC (Figure 7A). In contrast, we observed a notable increase in both NQO1 as well as Nrf2 levels. Conversely, the Nrf2 inhibitor ML385 effectively blocked the positive effects of EOSIR on these proteins. Similarly, silencing Nrf2 abolished the antioxidant and endothelial protective effects of EOSIR (Figure 7B). To study the impact of EOSIR and ML385 on ox-LDL-induced Nrf2 translocation in the nuclei of HUVECs, Nrf2 immunofluorescence staining was performed (Figure 7C). After the incubation of HUVECs with EOSIR, the nuclei displayed green fluorescence, indicating that Nrf2 was activated and translocated to the nucleus from the cytoplasm. The green fluorescence of Nrf2 in the nucleus was diminished after treatment with EOSIR and ML385, compared to the EOSIR group, indicating that ML385 inhibited the EOSIR-induced nuclear translocation of Nrf2 in HUVECs. Furthermore, the EOSIR and ML385 combination exerted an antagonistic effect. In summary, EOSIR enhances ox-LDL-stimulated activation of Nrf2 signaling in HUVECs.

## 4 Discussion

In this study, we examined the effects of EOSIR on VA and AS, as well as its underlying mechanisms, using both *ex vivo* and *in vivo* models. The results demonstrated that EOSIR treatment significantly improved blood lipid profiles and reduced the formation of arterial lipid plaques in a mouse model of AS. Moreover, EOSIR effectively inhibited ox-LDL-induced endothelial senescence, apoptosis, and mitochondrial damage in HUVECs by activating Nrf2 pathway. Taken together, these findings suggest that EOSIR holds promise as a therapeutic agent for delaying vascular aging and preventing or treating AS.

A key pathological mechanism of AS is the apoptosis of endothelial cells induced by ox-LDL. The process of apoptosis is accompanied by mitochondrial dysfunction, oxidative imbalance, and the upstream Bcl-2 family that regulates mitochondrial permeability and Cyt c release, etc. These events ultimately lead to DNA fragmentation and programmed cell death (Jan and Chaudhry, 2019). Ox-LDL triggers lipid accumulation, inflammatory responses, and further promotes endothelial cell apoptosis. Consequently, abnormal alterations in the levels of Bax and Bcl-2 can directly reflect the oxidative damage to endothelial cells (Bian et al., 2020). In the present study, we employed ox-LDL treatment of HUVECs to establish an AS model, demonstrating that ox-LDL enhanced the apoptosis rate of these cells, upregulated the pro-apoptotic factor Bax, and downregulated the anti-apoptotic factor Bcl-2. Notably, we also observed that EOSIR alleviated these effects, effectively inhibiting ox-LDL-induced apoptosis in HUVECs. The caspase family includes the initiator caspases (caspase-2, -8, -9, and -10) first activated by the apoptosis signal and the downstream effector caspases (caspase-3, -6, and -7) that execute apoptosis. These two groups play a dominant role in the initiation and execution of cell apoptosis (Boice and Bouchier-Hayes, 2020). Our results show that EOSIR decreased cleaved caspase-9 and cleaved caspase-3 expression, results that are consistent with typical features of apoptosis and with the regulation of mitochondria-dependent apoptosis. In summary, we conclude that EOSIR can improve apoptosis through the mitochondrial pathway.

Additionally, cellular senescence, oxidative stress, and impaired NO bioavailability contribute to the initiation or progression to endothelial dysfunction, thereby accelerating AS progression (Mikhed et al., 2015; Nowak et al., 2018). Oxidative stress induces growth arrest in endothelial cells, which subsequently triggers endothelial senescence. P53 and P21 are well-established markers of cellular senescence; elevated expression of these proteins leads to cell cycle arrest and eventually initiates programmed cell death (Gao et al., 2016). Our study demonstrated that EOSIR significantly reduced the rate of endothelial cell senescence induced by ox-LDL and notably decreased the expression of the P53 and P21 proteins. These findings were further supported by *in vivo* experiments, indicating that EOSIR effectively mitigates cellular senescence. These findings are aligned with the results previously reported by Prof. Chen (Chen et al., 2017).

Studies have demonstrated that senescent endothelial cells can accumulate in large quantities within the aortic wall, further contributing to endothelial dysfunction, damaging the vasculature, promoting plaque formation, and exacerbating a patient’s development in AS (Zhang et al., 2024; Song et al., 2023). Therefore, we also assessed the potential of EOSIR in ApoE^−/−^ mice in mitigating AS. The experimental results indicated that EOSIR improved lipid profiles and reduced the size of aortic plaques in these AS model mice. In summary, EOSIR reduces plaque formation, inhibits apoptosis and senescence of HUVECs in mice, and shows potential as a therapeutic candidate for AS.

Cellular stimulation induces an overproduction of ROS, and when these ROS are not neutralized in time, they trigger oxidative stress, causing subsequent cellular damage (Poznyak et al., 2022). Similar to NO, PGI2 exerts a vasodilatory effect and inhibits platelet aggregation. ET-1, a potent vasoconstrictor, interacts with PGI2 and other thromboxanes to maintain the balance between vascular systolic and diastolic function. This balance is disrupted when endothelial cells are exposed to oxidative stress, which can trigger vascular dysfunction and causing AS (Chirkov et al., 2022; Yang et al., 2019). In this study, we found that ox-LDL not only increased the production of cellular ROS but also elevated the levels of the vasoconstrictor factor ET-1 while reducing the levels of the vasodilators NO and PGI2. However, after EOSIR intervention, intracellular levels of NO and PGI2 were significantly increased, while ET-1 and ROS levels were significantly reduced. These results suggest that EOSIR exerts antioxidant and endothelial protective effects on HUVEC cells under ox-LDL-induced stress. Additionally, we observed that ML385, an Nrf2 inhibitor, effectively suppressed the beneficial effects of EOSIR on ROS, vasoactive factors (NO, PGI2, and ET-1), apoptosis, and senescence in HUVEC cells. This indicates that EOSIR may mitigate ox-LDL-induced cellular senescence and functional impairment by activating Nrf2.

Endothelial dysfunction caused by mitochondrial dysfunction is an important cause of AS. When mitochondrial dysfunction occurs, it decreases the mitochondrial membrane potential, hinders cellular energy production, leads to overproduction of ROS, and causes cellular senescence (Cadenas and Davies, 2000). In contrast, mitochondrial quality control mechanisms, including processes such as autophagy, fusion, and fission, help maintain mitochondrial stability. When cells are damaged, dysfunctional mitochondria are selectively eliminated, thereby preserving normal cellular function. Consequently, maintaining the balance of the mitochondrial quality control system is crucial for efficiently removing damaged mitochondria and delaying cellular senescence (Lin et al., 2021; Wu et al., 2019). Therefore, this paper further explored these effects of EOSIR in the quality control system of mitochondria. The results indicated that EOSIR significantly enhanced ATP production and MMP in HUVECs, mitigated the disruption of the mitochondrial network induced by ox-LDL, and reduced mitochondrial fragmentation. TEM analysis revealed that EOSIR restored mitochondrial morphology and reduced the formation of autophagic vesicles in HUVECs. Moreover, both *in vivo* and *in vitro* experiments demonstrated that EOSIR can elevate the levels of mitochondrial fusion proteins (MFN1, MFN2, and OPA1), mitochondrial biogenesis proteins (TFAM and PGC-1α), and p62, while reducing the expression of the mitochondrial fission protein Drp1 and the autophagy marker LC3II. Consequently, EOSIR appears to regulate mitochondrial homeostasis, mitigate oxidative stress-induced autophagic imbalance, and preserve endothelial function stability. Nrf2 has been identified as a key mediator of endogenous antioxidant defense and mitochondrial biogenesis, both of which are essential for regulating mitochondrial homeostasis and structural integrity (Cox et al., 2018). Similarly, after inhibiting Nrf2, we observed that EOSIR’s protective effect on the mitochondrial morphology and structure of HUVECs, as well as its regulation of related proteins, was also diminished. This result further supports the previous findings. In summary, EOSIR regulates mitochondrial quality control, mitigates cellular damage, and delays senescence, with this effect potentially linked to Nrf2 activation.

Nrf2 is a master regulator of the cellular redox response, maintaining low transcriptional activity in complex with Keap1. Upon stimulation by ROS, Nrf2 dissociates from Keap1 and translocates to the nucleus, where it binds to the ARE and activates the transcription of downstream antioxidant enzymes, thus mitigating oxidative damage (Cai et al., 2021). The Nrf2 signaling pathway plays a crucial role in regulating mitochondrial biogenesis and enhancing antioxidant defenses, thereby influencing AS through the modulation of downstream antioxidant factors. A variety of natural compounds like salvianolic acid and curcumin, have undergone extensive study, revealing their ability to activate Nrf2, thereby helping to slow the development of AS (Mao et al., 2018; Zhang et al., 2014). The research endeavored to uncover the mechanism EOSIR uses to alleviate AS, focusing on the inhibition and silencing of Nrf2. The results demonstrated that EOSIR could upregulate Nrf2, NQO1, and HO-1 expression while downregulating Keap1 in cells and mice. Furthermore, inhibition or silencing of Nrf2 reversed these effects of EOSIR, which is consistent with previous findings (Meng et al., 2022). Additionally, the immunofluorescence results revealed EOSIR markedly increased nuclear Nrf2 expression. In conclusion, the therapeutic mechanism of EOSIR in AS may involve the Nrf2 pathway’s activation and upregulation of downstream antioxidant factors.

## 5 Conclusion

This study demonstrated that EOSIR regulates the mitochondrial quality control system by activating the Nrf2 signaling pathway, significantly improving lipid profiles and reducing aortic plaque formation in an AS mouse model. It also effectively inhibited ox-LDL-induced senescence, apoptosis, and mitochondrial damage in HUVECs. Therefore, EOSIR holds potential as a therapeutic or preventive agent for AS, offering theoretical support for the use of Guangxi botanicals in treating cardiovascular diseases.

## Data Availability

The raw data supporting the conclusions of this article will be made available by the authors, without undue reservation.

## Abbreviations

AS, Atherosclerosis; BARD, Bardoxolone Methyl; DMSO, Dimethyl sulfoxide; Drp1, dynamin-related protein 1; EOSIR, essential oil from *Stahlianthus involucratus* rhizomes; HUVECs, Human umbilical vein endothelial cells; HO-1, Heme oxygenase 1; Keap1, Recombinant Kelch Like ECH Associated Protein 1; Mfn1, mitofusin 1; Mfn2, mitofusin 2; Nrf2, Nuclear E2-related factor 2; NQO1, NAD(P)H: quinone oxidoreductase 1; ox-LDL, oxidized low-density lipoprotein; Opa1, Optic Atrophy 1; Pink1, PTEN induced putative kinase 1; PGC-1α, peroxisome proliferatoractivated receptorγcoactivator-1α; PBS, Phosphate Buffered Saline; TFAM, mitochondrial transcription factor A.
