# Supplementary material for: The essential oil from the rhizomes of Stahlianthus involucratus attenuates the progression of vascular aging and atherosclerosis by regulating Nrf2-mediated mitochondrial quality
Source: Front Pharmacol. 2025 May 20;16:1579333. doi: 10.3389/fphar.2025.1579333 (PMC12130047; doi:10.3389/fphar.2025.1579333)
Supplement: Supplementary file 1 [file Table1.docx]

Supplementary Material

# Supplementary Tables

**Supplementary Table 1** Compounds detected in EOSIR by GC–MS. Data was expressed in relative percentages of area values (%).

| No. | t_R_/ min | Identification | Formula | Molecular weight | Content（%） |
| --- | --- | --- | --- | --- | --- |
| 1 | 9.759 | 2-Heptanol | C_7_H_16_O | 116 | 0.16 |
| 2 | 10.102 | Tricyclo [2.2.1.0(2,6)] heptane,1,7,7-trimethyl- | C_10_H_16_ | 136 | 0.42 |
| 3 | 10.56 | (1R)-2,6,6-Trimethylbicyclo [3.1.1] hept-2-ene | C_10_H_16_ | 136 | 5.29 |
| 4 | 11.211 | Camphene | C_10_H_16_ | 136 | 18.51 |
| 5 | 11.978 | p-Cymene | C_10_H_14_ | 134 | 0.22 |
| 6 | 12.172 | Bicyclo [3.1.1] heptane,6,6-dimethyl-2-methylene-, (1S)- | C_10_H_16_ | 136 | 0.11 |
| 7 | 12.749 | 5-Hepten-2-one, 6-methyl- | C_8_H_14_O | 126 | 0.03 |
| 8 | 13.331 | 3-Carene | C_10_H_16_ | 136 | 3.7 |
| 9 | 13.903 | Benzene, 1-methyl-3-(1-methylethyl)- | C_10_H_14_ | 134 | 0.52 |
| 10 | 14.047 | D-Limonene | C_10_H_16_ | 136 | 1.03 |
| 11 | 14.271 | Eucalyptol | C_10_H_18_O | 154 | 0.11 |
| 12 | 15.649 | α-Methyl-α-[4-methyl-3-pentenyl] oxiranemethanol | C_10_H_18_O_2_ | 170 | 0.04 |
| 13 | 15.843 | Benzene, 1-methyl-4-(1-methylethenyl)- | C_10_H_12_ | 132 | 0.04 |
| 14 | 16.042 | Bicyclo[2.2.1]heptan-2-one, 3,3-dimethyl- | C_9_H_14_O | 138 | 0.11 |
| 15 | 16.276 | 2-Nonanone | C_9_H_18_O | 142 | 0.06 |
| 16 | 16.595 | 1,6-Octadien-3-ol, 3,7-dimethyl- | C_10_H_18_O | 154 | 0.47 |
| 17 | 16.764 | 2-Nonanol | C_9_H_20_O | 144 | 0.8 |
| 18 | 17.371 | α-Campholenal | C_10_H_16_O | 152 | 0.06 |
| 19 | 17.615 | 3-Cyclohexene-1-carboxaldehyde, 1,3,4-trimethyl- | C_10_H_16_O | 152 | 0.12 |
| 20 | 18.142 | (+)-2-Bornanone | C_10_H_16_O | 152 | 19.49 |
| 21 | 18.296 | Bicyclo[2.2.1]heptan-2-ol, 2,3,3-trimethyl- | C_10_H_18_O | 154 | 1.09 |
| 22 | 18.52 | Pinocarvone | C_10_H_14_O | 150 | 0.04 |
| 23 | 19.525 | α-Terpineol | C_10_H_18_O | 154 | 0.67 |
| 24 | 19.719 | Bicyclo[3.1.1]hept-2-ene-2-methanol, 6,6-dimethyl- | C_10_H_16_O | 152 | 0.77 |
| 25 | 20.003 | Bicyclo[3.1.1]hept-3-en-2-one, 4,6,6-trimethyl- | C_10_H_14_O | 150 | 0.42 |
| 26 | 20.411 | Bicyclo[2.2.1]heptan-2-ol, 7,7-dimethyl-, acetate | C_11_H_18_O_2_ | 182 | 0.11 |
| 27 | 20.575 | 2-Acetoxydodecane | C_14_H_28_O_2_ | 228 | 0.05 |
| 28 | 21.112 | D-Verbenone | C_10_H_14_O | 150 | 0.05 |
| 29 | 21.291 | Ethanol, 2-(3,3-dimethylcyclohexylidene)-, (Z)- | C_10_H_18_O | 154 | 0.02 |
| 30 | 22.043 | Bornyl acetate | C_12_H_20_O2 | 196 | 0.09 |
| 31 | 22.247 | 2-Undecanone | C_11_H_22_O | 170 | 0.02 |
| 32 | 23.729 | Tricyclo[5.4.0.0(2,8)]undec-9-ene, 2,6,6,9-tetramethyl-, (1R,2S,7R,8R)- | C_15_H_24_ | 204 | 0.06 |
| 33 | 23.854 | α-Guaiene | C_15_H_24_ | 204 | 0.09 |
| 34 | 24.232 | 1,2,4-Metheno-1H-indene, octahydro-1,7a-dimethyl-5-(1-methylethyl)-, [1S-(1α,2α,3aβ,4α,5α,7aβ,8S*)]- | C_15_H_24_ | 204 | 0.11 |
| 35 | 24.451 | alfa-Copaene | C_15_H_24_ | 204 | 9.06 |
| 36 | 25.207 | Aromadendrene, dehydro- | C_15_H_22_ | 202 | 0.09 |
| 37 | 25.525 | Tricyclo[2.2.1.0(2,6)]heptane, 1,7-dimethyl-7-(4-methyl-3-pentenyl)-, (-)- | C_15_H_24_ | 204 | 0.16 |
| 38 | 25.814 | 1H-Cyclopenta [1,3] cyclopropa [1,2] benzene, octahydro-7-methyl-3-methylene-4-(1-methylethyl)-, [3aS-(3aα,3bβ,4β,7α,7aS*)]- | C_15_H_24_ | 204 | 0.05 |
| 39 | 26.038 | 1H-Cycloprop[e]azulene, decahydro-1,1,7-trimethyl-4-methylene- | C_15_H_24_ | 204 | 0.03 |
| 40 | 26.341 | 1H-Benzocycloheptene, 2,4a,5,6,7,8,9,9a-octahydro-3,5,5-trimethyl-9-methylene-, (4aS-cis)- | C_15_H_24_ | 204 | 0.05 |
| 41 | 26.61 | Alloaromadendrene | C_15_H_24_ | 204 | 4.18 |
| 42 | 26.948 | γ-Muurolene | C_15_H_24_ | 204 | 0.82 |
| 43 | 27.088 | Benzene, 1-(1,5-dimethyl-4-hexenyl)-4-methyl- | C_15_H_22_ | 202 | 0.4 |
| 44 | 27.307 | Naphthalene, decahydro-4a-methyl-1-methylene-7-(1-methylethenyl)-, [4aR-(4aα,7α,8aβ)]- | C_15_H_24_ | 204 | 0.44 |
| 45 | 27.481 | Naphthalene, 1,2,3,4,4a,5,6,8a-octahydro-4a,8-dimethyl-2-(1-methylethenyl)-, [2R-(2α,4aα,8aβ)]- | C_15_H_24_ | 204 | 0.17 |
| 46 | 27.884 | Naphthalene, 1,2,3,4,4a,5,6,8a-octahydro-7-methyl-4-methylene-1-(1-methylethyl)-, (1α,4aβ,8aα)- | C_15_H_24_ | 204 | 0.36 |
| 47 | 28.008 | Naphthalene, 1,2,4a,5,8,8a-hexahydro-4,7-dimethyl-1-(1-methylethyl)-, [1S-(1α,4aβ,8aα)]- | C_15_H_24_ | 204 | 0.39 |
| 48 | 28.103 | Naphthalene, 1,2,3,4-tetrahydro-1,6-dimethyl-4-(1-methylethyl)-, (1S-cis)- | C_15_H_22_ | 202 | 0.84 |
| 49 | 28.421 | Isolongifolene, 4,5,9,10-dehydro- | C_15_H_20_ | 200 | 0.08 |
| 50 | 28.58 | α-Calacorene | C_15_H_20_ | 200 | 0.59 |
| 51 | 29.078 | α-Calacorene | C_15_H_20_ | 200 | 0.1 |
| 52 | 29.197 | Isoshyobunone | C_15_H_24_O | 220 | 0.04 |
| 53 | 29.635 | Caryophyllene oxide | C_15_H_24_O | 220 | 2.69 |
| 54 | 29.829 | Tricyclo [5.2.2.0(1,6)] undecan-3-ol, 2-methylene-6,8,8-trimethyl- | C_15_H_24_O | 220 | 0.82 |
| 55 | 30.083 | 1,4-Methanoazulen-9-one, decahydro-1,5,5,8a-tetramethyl-, [1R-(1α,3aβ,4α,8aβ)]- | C_15_H_24_O | 220 | 0.32 |
| 56 | 30.272 | 12-Oxabicyclo [9.1.0] dodeca-3,7-diene, 1,5,5,8-tetramethyl-, [1R-(1R*,3E,7E,11R*)]- | C_15_H_24_O | 220 | 1.27 |
| 57 | 30.963 | tau-Cadinol | C_15_H_26_O | 222 | 0.21 |
| 58 | 31.282 | 2-Naphthalenemethanol, 1,2,3,4,4a,5,6,8a-octahydro-α, α,4a,8-tetramethyl-, [2R-(2α,4aα,8aβ)]- | C_15_H_26_O | 222 | 0.4 |
| 59 | 31.62 | Naphthalene, 1,6-dimethyl-4-(1-methylethyl)- | C_15_H_18_ | 198 | 0.28 |
| 60 | 32.68 | 2(1H) Naphthalenone, 3,5,6,7,8,8a-hexahydro-4,8a-dimethyl-6-(1-methylethenyl)- | C_15_H_22_O | 218 | 0.04 |
| 61 | 32.794 | Isoaromadendrene epoxide | C_15_H_24_O | 220 | 0.03 |
| 62 | 33.784 | 2,4,6-Cycloheptatrien-1-one, 2-hydroxy-5-(3-methyl-2-butenyl)-4-(1-methylethenyl)- | C_15_H_18_O_2_ | 230 | 0.62 |
| 63 | 34.675 | As-Indacen-1(2H)-one, 3,6,7,8-tetrahydro-3,3,6,6-tetramethyl- | C_16_H_20_O | 228 | 20.52 |
| 64 | 36.292 | As-Indacen-1(2H)-one, 3,6,7,8-tetrahydro-3,3,6,6-tetramethyl- | C_16_H_20_O | 228 | 0.07 |
